# The association between oral microbiome and gastric precancerous lesions

**DOI:** 10.1128/msystems.01322-24

**Published:** 2024-12-04

**Authors:** Yifei Chen, Lei Lei, Mengying Xia, Ran Cheng, He Cai, Tao Hu

**Affiliations:** 1 State Key Laboratory of Oral Diseases & National Center for Stomatology & National Clinical Research Center for Oral Diseases & Frontier Innovation Center for Dental Medicine Plus, West China Hospital of Stomatology, Sichuan University, Chengdu, Sichuan, China; Istanbul Medipol University School of Medicine, Istanbul, Turkey

**Keywords:** oral microbiome, gastric precancerous lesions, atrophic gastritis, intestinal metaplasia

## Abstract

Gastric precancerous lesions are thought to be precursors in the occurrence and development of gastric cancer through Correa's cascade. Recent studies have investigated the association between the oral microbiome and gastric precancerous lesions. However, there has yet to be a comprehensive synthesis review of the existing literature on the relationship between oral microbiome and gastric precancerous lesions. A systematic review was conducted to characterize the literature on the association between oral microbiome and gastric precancerous lesions. The studies show that oral microbiome is dynamic in individuals with gastric precancerous lesions. Oral-derived microorganisms were colonized in the gastric precancerous lesions. Interactions between oral and gastric microbiomes affect the response of the host immunity. The abnormal proliferation of oral-associated microorganisms may be linked to the reduction of gastric acid. The present review supports the potential association between oral microbiome and gastric precancerous lesions. However, the interactions are complex and multifaceted, which require further investigation.

## INTRODUCTION

According to the GLOBOCAN 2020 estimates of cancer incidence and mortality, gastric cancer (GC) belongs to one of the most common and fatal malignancy in the world (1). Gastric carcinogenesis is a multifactorial chronic process related to the interaction between the host genetic susceptibility and various environmental factors, resulted in chronic gastritis, cellular atrophy, and the degeneration of intrinsic glands, intestinal metaplasia (replacement of gastric epithelium with intestinal epithelium) of gastric mucosal epithelial cells, dysplasia (intraepithelial neoplasia), and sequentially, gastric carcinoma (2). The progressive histopathological changes in the gastric mucosa before GC are gastric precancerous lesions. According to the Correa's cascade model and clinical examination experience, gastric precancerous lesions can be categorized into three stages: atrophic gastritis (AG), intestinal metaplasia (IM), and intraepithelial neoplasia (3). *Heliobacter pylori* (*H. pylori*) is one of the key carcinogenicity factors in the gastric microbiome that can influence gastric precancerous lesions. In addition to *H. pylori*, the genera *Streptococcus*, *Acinetobacter*, and *Hemophilus* located in gastric mucosa are also involved in the progression of gastric precancerous lesions (3). The changes in the gastric microbiome identified associations between gastric precancerous lesions and microorganisms. The oral cavity is the second largest microbial ecosystem within the human body, following the intestinal tract. It is biologically connected to the intestine, with oral microbiota entering the gastrointestinal tract via the mucosal barrier and esophagus (4, 5). The dysbiosis in oral and intestinal microbiota may contribute to gastrointestinal diseases through immune regulation and inflammatory responses (6, 7). Since inflammatory cytokines can regulate gastric carcinogenesis, there may be a potential link between oral microorganisms and gastric precancerous lesions (8). However, there are controversial results existing in these studies. In the previous study, an oral microbial infectious disease, periodontitis, was positively associated with gastric precancerous lesions (9). Correspondingly, periodontal *Prevotella* pathogens were significantly enriched in the stomach of AG cases. However, not all periodontal pathogens are positively associated with it (10). In contrast, another periodontal pathogen, *Tannerella forsythia* (*T. forsythia*), was negatively correlated with the progression of gastric precancerous lesions (11). Recent studies have investigated the oral microbiome in patients with gastric precancerous lesions, suggesting that oral-originated microbiota might also be involved in gastric carcinogenesis procedure. Thus, the present review summarized the association between oral microorganisms and gastric precancerous lesions with some potential mechanisms being also discussed.

## EPIDEMIOLOGICAL EVIDENCE ASSOCIATED ORAL MICROBIOME TO GASTRIC RECANCEROUS LESIONS

An increasing amount of evidence suggested that oral microbiome is correlated with gastric precancerous lesions. The main characteristics and findings of the studies were shown in Table 1. Thirteen studies were published between 2013 and 2024 and were consisted of seven case-control studies, two cohort studies, and four cross-sectional studies. As for the sample sites, seven studies were sampled in the oral cavity (saliva, dental plaque, tongue coating, and periodontal tissue), while six were sampled in the gastrointestinal tract (gastric mucosa, gastric juices, duodenal mucosa, and feces).

**TABLE 1:** Characteristics and findings of the epidemiological evidence

Author (year)	Dominant genes/species	Country, study design	Gastric precancerous lesion cases	Controls	Sample source	Microbiology analysis	Outcomes of interest
Liu et al. (2023) (12)	*Alloprevotella* and *Streptococcus*	China, case-control study	53 inflammation cases (female, 50.94%; age, 47 [37–53]); 31 AG cases (female, 45.16%; age, 51 [40–44, 47–60]); 49 IM cases (female, 57.14%; age, 54 [47–61.5])	NA	Tongue coating	16S rRNA gene sequencing and random forest analysis	*Alloprevotella* and *Streptococcus* were higher in IM with greasy coating. *Alloprevotella* was a potential oral biomarker to diagnose IM with the AUC value of 0.74.
Chen et al. (2022) (11)	*F. nucleatum*	China, case-control study	31 AG cases (female, 45.16%; age, 62.13 ± 10.56)	20 healthy controls (female, 7; age, 56.90 ± 16.02)	Whole saliva	Droplet digital PCR for bacterial strains	*F. nucleatum* level in AG was higher than that in normal controls.
Wu et al. (2022) (13)	*Peptostreptococcus stomatis*, *Johnsonella ignava*, *N. elongata*, and *N. flavescens*	United States, case-control study	89 IM cases (female, 58.4%; age, 58.3 ± 10.9)	89 healthy controls (female, 58.4%; age, 57.3 ± 10.5)	Saliva and oral wash samples	Metagenomic sequencing	Oral bacterial species *Peptostreptococcus stomatis*, *Johnsonella ignava*, *N. elongata*, and *N. flavescens* were enriched in patients with IM.
Huang et al. (2021) (14)	*Anaerovorax*, *Bulleidia*, unclassified F16, *Peptostreptococcus*, *Bacteroides* genus, and *Prevotella*	China, cross-sectional study	21 AG cases without IM (female, 52.4%; age, 49.9 ± 12.5); 72 AG with IM (female, 45.8% ; age, 48.5 ± 11.7)	10 SG cases (female, 49.5%; age: 48.2 ± 10.2); 99 GC cases (female, 35.4%; age: 49.6 ± 8.8)	Saliva	16S rRNA gene sequencing and network analysis	*Anaerovorax, Bulleidia,* unclassified F16, and *Peptostreptococcus* decreased from SG through AG to GC. *Bacteroides* genus was more abundant in the AG cases. *Prevotella* was negatively associated with a variety of oral bacteria.
Luo et al. (2020) (15)	*H. pylori*	China, case-control study	30 periodontitis patients with AG (female, 50%; age, 51.4 ± 12.7)	35 periodontitis patients without AG (age, 47.5 ± 10.5; female, 57.1%)	Periodontitis tissues	Microbial DNA Quantitative PCR Multi-Assay H. pylori Kit	In contrast to the non-AG group, the *H. pylori* the infection rate of periodontal tissues in the AG group increased.
Cui et al. (2019) (16)	*C. concisusin*	China, case-control study	11 AG cases (female, 72.7%; age, 47 ± 12.3); 23 IM cases (female, 56.5%; age, 55 ± 11.5)	50 healthy controls (female, 54.0%; age, 44 ± 15.6)	Tongue coating	Metagenomic sequencing with the Illumina HiSeq platform	*C. concisus* enriched during the progression of the precancerous cascade (typical to AG to IM)
Salazar et al. (2013) (17)	*T. forsythia*, *A. actinomycetemcomitans*	United States, cross-sectional study	37 cases of AG, IM, or dysplasia (female, 67.6%; age: 58.2 ± 9.0)	82 healthy controls (female, 61.0%; age, 56.9 ± 9.0)	Plaque and saliva samples	Quantitative real-time PCR	In saliva, *T. forsythia* was inversely associated with gastric precancerous lesions. In plaque, *A.actinomycetemcomitans* were positively associated with gastric precancerous lesions among patients with more severe periodontal disease.
Hua et al. (2023) (18))	*Neisseria*, *Staphylococcus*, *Haemophilus*, *Veillonella*, *Prevotella* 7, and *Rothia*	China, cross-sectional study	124 AG cases (female, 54.0%; age, 58.4 ± 7.2)	69 non-AG chronic gastritis cases (female, 56.5%; age: 55.7 ± 6.9)	Gastric sinus mucosa	16S rRNA gene sequencing	Oral microbiota, such as genus *Neisseria*, *Staphylococcus*, *Haemophilus* decreased in the HP-I CAG cases. Bile reflux promoted the colonization of oral microbiota (*Veillonella*, *Prevotella* 7, and *Rothia*) in the CAG cases
Dong et al. (2022) (19)	*Porphyromonas gingivalis*, *Campylobacter gracilis*, and *Granulicatella elegans*	China, case-control study	20 AG cases (female, NA; age, NA)	30 non-AG cases (female, NA; age: NA)	Gastric juices	16S rRNA gene sequencing	The compositional analysis of "core microbiota” revealed that oral pathogens, including *Porphyromonas gingivalis*, *Campylobacter gracilis*, and *Granulicatella elegans*, were abundant in AG cases
Ndegwa et al. (2020) (10)	*Peptostreptococcus*, *Prevotella*, *Centipeda*, *Actinomyces*, and *Atopobium*	Sweden, cross-sectional study	12 AG cases (female, 50%; age, 69.25 [7.3])	171 healthy cases (female: 49.7%; age, 51.92 [14.1])	Gastric mucosa	PCR and Microbial co-occurrence network analysis	The positive co-occurrence of oral pathogens including *Peptostreptococcus*, *Prevotella*, *Centipeda*, *Actinomyces*, and *Atopobium* increased in the stomach.
Sung et al. (2020) (20)	*Peptostreptococcus*, *Streptococcus*, *Parvimonas*, *Rothia*, and *Granulicatella*	China, cohort study	102 *H. pylori*–positive cases of inflammation, AG, and IM before and after 1 year *H. pylori* eradication (female, 48.1%; age, 52.46 [7.18])	100 *H. pylori*–positive cases of inflammation, AG, and IM before and after 1 year placebo (female, 54.2%; age, 52.24 [6.4])	Gastric mucosa	16S rRNA gene sequencing and quantitative PCR.	*Peptostreptococcus* were positively correlated with the atrophy scores. The enrichment of *Granulicatella*, *Streptococcus*, and *Rothia* was in patients of emerged atrophy 1 year after *H. pylori* eradication. *Peptostreptococcus* and *Parvimonas* were enriched in patients with progressed or persisted IM.
Filardo et al. (2022) (21)	*Rothia mucilaginosa, Granulicatella adiacens*, and *Streotococcus salivarius*	Italy, case-control study	20 AG cases (female, 75%; age, 52.2 ± 13.1)	11 healthy cases (female, 91%; age, 51 ± 13.5)	Duodenal mucosa	16s rRNA gene sequencing	Chronic AG was associated to the oral bacteria, such as *Rothia mucilaginosa*, *Streptococcus salivarius*, and *Granulicatella adiacens*.
Furune et al. (2022) (22)	*Actinomyces*, *Aggregatibacter*, *Campylobacter*, *Granulicatella*, *Pyramidobacter*, *Streptococcus*, *Cardiobacterium*, and *Haemophilus*	Japan, prospective cohort study	5 progressive AG cases (female, 20%; age, 62.5 [43, 44, 47–77]); 10 non-progressive AG cases (female, 40%; age, 74 [67–85])	NA	Feces	Targeting 16S rRNA using Illumina Miseq	At 8 weeks after eradication, 12 genera showed a significant difference in the relative abundance between progressive AG and non-progressive AG, among which eight genera, namely, *Actinomyces*, *Aggregatibacter*, *Campylobacter*, *Granulicatella*, *Pyramidobacter*, *Streptococcus*, *Cardiobacterium*, and *Haemophilus* were oral-derived bacteria.

^*a*^ AG: atrophic gastritis; IM: intestinal metaplasia; SG: superficial gastritis; NA: non-applicable.

## ORAL MICROBIOME IN PATIENTS WITH GASTRIC PRECANCEROUS LESIONS

In the present studies, oral microbiomes from saliva, dental plaque, tongue coating, and periodontal tissue were sampled in the oral cavity. Distinct oral microbiomes in different regions of the oral cavity are linked to gastric precancerous lesions. The salivary microbiome consists of the dissociative microbiome in the oral cavity, which remains relatively constant and interacts with the intestinal microbiome (23, 24). Saliva was used to identify alterations of the oral microbiome in patients of gastric precancerous lesions. A cross-sectional study of saliva microbiota utilizing 16S rRNA sequencing revealed notable shifts of microbial genera across different gastric precancerous lesion stages. *Anaerovorax*, *Bulleidia*, unclassified F16, and *Peptostreptococcus* were decreased in abundance from superficial gastritis (SG), AG to GC, while *Bacteroides* was more abundant in AG than SG and GC (14). Another case-control study of saliva and oral wash by metagenomic sequencing explored the relationship between oral microbiome and IM. *Peptostreptococcus stomatis*, *Johnsonella ignava*, *Neisseria elongata* (*N. elongata*)*,* and *Neisseria flavescens* (*N. flavescens*) were enriched in patients with IM (13). It suggested that the salivary microbiome has the potential to predict gastric precancerous lesions. Currently, many studies indicated a close relationship among tongue coating, tongue microorganisms, and gastric diseases (25, 26). Likewise, it has been confirmed that tongue coating and tongue coating microorganisms are also associated with precancerous lesions of the stomach. Recent studies showed that tongue coating was associated with gastric precancerous lesions. A case-control study (16) assessed microorganisms in tongue coating and revealed a higher abundance of *Campylobacter concisus* (*C. concisus*) in patients with AG and IM than in healthy individuals. *C. concisus* is a naturally colonized bacteria in the oral cavity, which may induce inflammation and host immune responses associated with gastrointestinal diseases such as inflammatory bowel disease (27, 28). Additionally, *C. concisus* was detected in tongue and gastric juice in patients with gastritis, showing a high prevalence oforal *C. concisus* with gastric precancerous lesions (16). Meanwhile, a perspective suggested a correlation between the color and texture of the tongue coating and gastric mucosal lesions in the development of chronic AG (29, 30). A case-control study showed that greasy coating was significantly related to IM. According to the 16s rRNA gene sequencing results, *Streptococcus*, *norank_p__Saccharibacteria*, *Alloprevotella*, *Atopobium*, *Megasphaera*, unclassified_f__Prevotellaceae, *Gemella*, *Moraxella*, *Solobacterium*, and *Stomatobaculum* were higher abundance in greasy coating than non-greasy coating. Alloprevotella might be a potential oral biomarker to diagnose IM, with the value of area under receiver operating characteristic curve reached 0.74 (12). Regarding the periodontal pathogens elevated in patients with gastric precancerous lesions, *Fusobacterium nucleatum* (*F. nucleatum*) is recognized for promoting the development and metastasis of digestive tumors (31, 32). Chen et al. (11) found that salivary *F. nucleatum* was significantly abundant in patients with AG compared to the healthy cases. Some periodontal pathogens, *Porphyromonas gingivalis*, *T. forsythia*, *Treponema denticola*, and *Aggregatibacter actinomycetemcomitans* (*A. actinomycetemcomitans*) were also studied. *T. forsythia* in saliva showed an inverse correlation with gastric precancerous lesions. Furthermore, *A. actinomycetemcomitans* in dental plaque showed a positive association with gastric precancerous lesions and severe indicators of periodontal diseases, including clinical attachment loss, periodontal probing depth, and bleeding on probing. The combined status of periodontitis and corresponding periodontal pathogens may contribute to the risk of gastric precancerous lesions (17). Likewise, AG-associated abnormal proliferation of *H. pylori* in the oral microbiomes may also affect periodontitis. Patients with periodontitis and AG exhibited a significantly higher prevalence of *H. pylori* infections in periodontitis tissues than those without AG (15). Since oral *H. pylori* was found to aggravate the progress of periodontitis (33, 34), periodontitis patients with AG may get worse owing to the increased oral *H. pylori*. The above studies indicated that patients with gastric precancerous lesions have some reflection of the oral microbiome. Inversely, as the oral microbiome can specifically survive and reside in the stomach due to anatomical connection (35), the interaction and relationship between oral-derived microbiome and the underlying influence on the gastric precancerous lesions need to be discussed.

## ORAL-DERIVED MICROBIAL INVOLVEMENT IN GASTRIC PRECANCEROUS LESIONS

The oral-derived microbiome can potentially migrate and colonize in the stomach across the digestive tract. Gastric precancerous lesions influence the compositions of colonized oral-derived microbiome. Dong et al. (19) observed a manifest decrease in the diversity of microorganisms in the gastric juices of AG patients compared to those with non-AG. Core microbiota analysis revealed an amplification of oral pathogens such as *P. gingivalis*, *Campylobacter gracilis*, and *Granulicatella elegans* in AG cases (19). Another study (10) found that the oral pathogen *Prevotella* was significantly enriched in the stomach of AG cases. Additionally, the mutual exclusivity was verified between oral-derived microbiome and nonoral microbiome in the stomach. Compared with healthy cases, the stronger positive relationship between the oral-derived microbiome and AG illustrated the symbiosis and co-occurrence pattern over oral-derived microbiome, including *Prevotella*, *Peptostreptococcus*, *Centipeda*, *Actinomyces*, and *Atopobium* (10, 36). Nevertheless, the strongest negative correlation occurred between oral and non-oral bacteria communities, indicating the mutual exclusion between oral and non-oral bacteria. Thus, with the relative abundance of oral microbiome increasing in AG, nonoral bacteria may be excluded by oral bacteria (10, 36). The mutual exclusivity is especially observed between oral-derived microbiome and *H. pylori*. The principal cause of most chronic AG is *H. pylori* infection (37). Based on toxicity, gastric *H. pylori* infection can be classified into virulent Hp-I infection (carcinogenic) and avirulent Hp-II infection (38, 39). A case-control study showed that oral-derived microbiome such as genus *Neisseria*, *Staphylococcus*, and *Haemophilus* was significantly decreased in Hp-I CAG patients (18).

A small number of unexplained non-*H. pylori* AG implies that non-*H. pylori* microbiota in the stomach may also affect AG (40, 41). An excess risk of GC was observed in a low-*H. pylori* prevalent population, indicating that non-*H. pylori* microbiota may be involved through the Correa's cascade to GC (40). A recent study demonstrated that oral-associated microbiota was more abundant in GC located in the upper third of the stomach while *H. pylori* dominating in GC occurred in the lower third of the stomach (42). In a cohort study exploring the changes of the gastric microorganisms 1 year after *H. pylori* eradication, it was identified that the presence of oral microbiota, including *Peptostreptococcus*, *Streptococcus*, *Parvimonas*, *Rothia*, and *Granulicatella*, was significantly correlated to the persistence of AG and IM. *Peptostreptococcus* was positively associated with the severity of AG, while the enrichment of *Granulicatella*, *Streptococcus*, and *Rothia* was observed in patients with emerged atrophy after *H. pylori* eradication. Moreover, *Peptostreptococcus* and *Parvimonas* were abundant in patients with progressed or persisted IM (20). Oral-associated microbiota might be contributed to non-*H. pylori* induced gastric precancerous lesions.

## ORAL-GUT MICROBIOME AXIS IN GASTRIC PRECANCEROUS LESIONS

Furthermore, oral microbiome and gut microbiome are closely connected, and their interaction is termed the oral-gut axis (43). Under normal physiological conditions, they can maintain a fine-tuned balance. However, the occurrence and development of gastrointestinal disorders may alter the balance of the microecology of the oral-gut axis (44). Filardo et al. (21) revealed that increases in several oral-derived bacteria among the duodenal microbiota, including *Rothia mucilaginosa*, *Granulicatella adiacens*, and *Streptococcus salivarius*, were significantly correlated with chronic AG. Similarly, Furune et al. (22) compared fecal microbiome in AG before and after *H. pylori* eradication. Specifically, eight oral genera, including *Actinomyces*, *Aggregatibacter*, *Campylobacter*, *Granulicatella*, *Pyramidobacter*, *Streptococcus*, *Cardiobacterium*, and *Haemophilus*, showed higher relative abundance in progressive AG with atrophy extending beyond the cardia than non-progressive AG with confined atrophy. After *H. pylori* eradication*,* oral-derived bacteria in gut microbiome showed no significant difference in the relative abundance between progressive AG and non-progressive AG. The studies suggested a connection between the microbial dysbiosis and gastric precancerous lesions in the presence of the oral-gut axis.

## ORAL-DERIVED MICROBIOME ALTERATION IN GASTRIC PRECANCEROUS LESIONS

**Fig 1:**
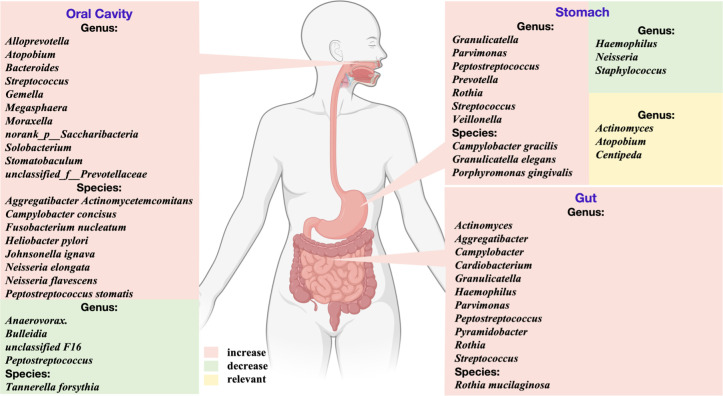
Changes of the associated oral microbiome in gastric precancerous lesions. In the oral cavity, *Bacteroides, Aggregatibacter actinomycetemcomitans, Campylobacter concisus, Fusobacterium nucleatum, Peptostreptococcus stomatis*, *Johnsonella ignava*, *Neisseria elongata*, and *Neisseria flavescens* were positively associated with gastric precancerous lesions. *Anaerovorax*, *Bulleidia*, unclassified F16, *Peptostreptococcus*, and *Tannerella forsythia* negatively related to gastric precancerous lesions. In the gastrointestinal tract, *Prevotella, Parvimonas, Peptostreptococcus, Granulicatella*, *Streptococcus*, *Rothia*, *Porphyromonas gingivalis*, *Campylobacter gracilis*, and *Granulicatella elegans* were positively associated with gastric precancerous lesions (figure created with BioRender.com).

The microbiome in the oral cavity and oral-derived microbiome in the gastrointestinal tract appeared to alter with the development of gastric precancerous lesions. There are positive and negative correlations between specific oral microorganisms and the progression of gastric lesions (Fig. 1). In the oral cavity, the periodontal pathogens *F. nucleatum* is enriched in patients with AG (11). *T. forsythia* is negatively correlated to gastric precancerous lesions, while *A. actinomycetemcomitans* showed a positive correlation (17). Meanwhile, AG could increase the prevalence of *H. pylori* in periodontitis tissues (33). During the precancerous cascade, *C. concisus* and *Bacteroides* exhibited enrichment, whereas *Anaerovorax*, *Bulleidia*, unclassified F16, and *Peptostreptococcus* decreased in abundance (14, 16). Besides, *Peptostreptococcus stomatis*, *Johnsonella ignava*, *N. elongata*, and *N. flavescens* were enriched in cases of gastric IM (13). In particular, *Streptococcus*, *norank_p__Saccharibacteria*, *Alloprevotella*, *Atopobium*, *Megasphaera*, unclassified_f_Prevotellaceae, *Gemella*, *Moraxella*, *Solobacterium*, and *Stomatobaculum* were increased in IM patients with greasy coating than those without non-greasy coating. Among them, *Alloprevotella* was significantly positively correlated with IM (12). These findings highlight a complex interplay between oral microbiome and gastric precancerous lesions, with both positive and negative correlations identified.

In the gastrointestinal tract, various oral-derived microorganisms also have a complex relationship with gastric precancerous lesions. In the stomach, *P. gingivalis*, *Campylobacter gracilis*, and *Granulicatella elegans* were positively associated with gastric precancerous lesions (19). Additionally, oral bacteria such as *Peptostreptococcus, Prevotella, Centipeda, Actinomyces*, and *Atopobium* exhibited dominance in relative abundance among the gastric microbiomes of AG (10). In chronic atrophic gastritis (CAG) cases with HP-I infection, oral microbiota such as *Neisseria, Staphylococcus*, and *Haemophilus* decreased, while bile reflux promoted the colonization of oral bacteria like *Veillonella, Prevotella*, and *Rothia* (18). Moreover, owing to the oral-gut axis, oral-derived *Rothia mucilaginosa* in gut microbiomes were positively correlated with AG (21). Various oral bacteria were also involved in AG, which is more prone to carcinogenesis. In the gut, oral-derived bacteria, including *Actinomyces, Aggregatibacter, Campylobacter, Granulicatella, Pyramidobacter, Streptococcus, Cardiobacterium*, and *Haemophilus* have increased in progressive AG cases with larger atrophy area (22). Generally, *H. pylori* is a significant risk factor for gastric carcinogenesis (45). However, AG cases without *H. pylori* exhibit an elevated risk of developing GC (40). In patients with emerged AG after *H. pylori* eradication, the enrichment of oral bacteria *Granulicatella, Streptococcus*, and *Rothia* was observed. *Peptostreptococcus* and *Parvimonas* were abundant in patients with progressed or persisted IM after *H. pylori* eradication (20). The continued progression of AG after *H. pylori* eradicating may be related to the shift in oral microbiomes. These findings emphasize the comprehensive relationship between oral-associated microbiome and gastric precancerous lesions in the gastrointestinal tract.

## MECHANISM OF ORAL MICROBIOME'S LOCATION IN GASTRIC PRECANCEROUS LESIONS

**Fig 2:**
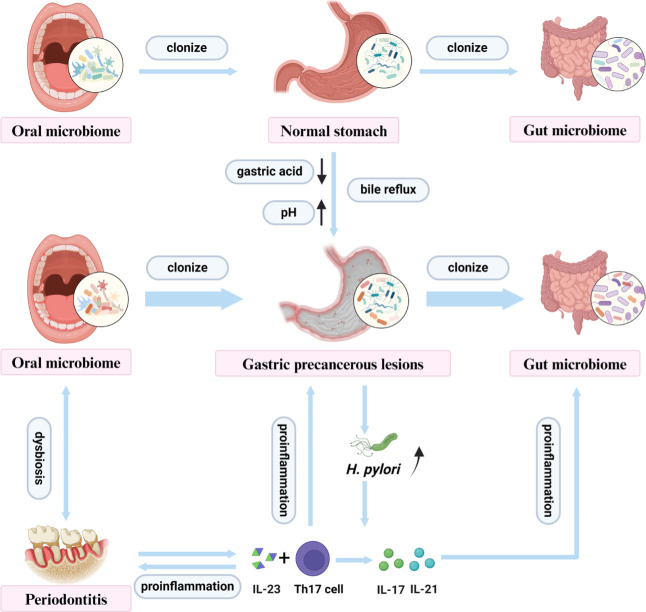
Mechanism of correlation between oral microbiome and gastric precancerous lesions. *Heliobacter pylori* and oral microbiome dysbiosis increased the levels of Th17 cells and their effector cytokines IL-17, IL-23, and IL-21, which accelerated pathogenesis of enteritis. Reduced gastric acid and bile reflux in the stomach lead to abnormal colonization of oral microorganisms. The two mechanisms accelerate inflammation and proliferation of oral microbiome (figure created with BioRender.com).

For the potential association between gastric precancerous lesions and oral microbiome, several studies have also attempted to explore the interaction mechanism between the two. In Fig. 2, two possible mechanisms have been exhibited.

*H. pylori* and oral microbiome dysbiosis increased the levels of Th17 cells and their effector cytokines IL-17, IL-23, and IL-21, which accelerated pathogenesis of enteritis. Reduced gastric acid in the stomach leads to abnormal colonization of oral microorganisms. The two mechanisms accelerate inflammation and proliferation of oral microbiome.

Interactions between oral and gastrointestinal microbiomes may affect the response of the host immune system. Compared to the non-chronic AG, chronic AG group showed a significantly higher prevalence of *H. pylori* infection and increased levels of Th17 cell subsets and cytokines (IL-17, IL-23, and IL-21) (15). The immune response can be activated after IL-23 enables naive CD4+ T cells into Th17 cells and to secret their effector cytokines, such as IL-17 and IL-21 (15). Furthermore, oral microbiome dysbiosis caused by periodontitis induces the production of Th17 cells, which migrate to the intestine and undergo expansion in response to bacterial antigens, thereby accelerating the pathogenesis of enteritis (4). Th17 cell subsets and cytokines play an essential role in the immune response and are closely associated with infection control and the preneoplastic stage (46). It can be hypothesized that Th17 cells may interact with oral microbiome and gastric precancerous lesions, especially AG.

The colonization and proliferation of oral-derived microbiomes was related to the environmental shifts, such as reduced gastric acid secretion and Bile reflux. The reduced gastric acid secretion, resulting from *H. pylori* infection (47) or gastric precancerous lesions may lead to abnormal proliferation of oral microorganisms in the stomach (21, 48). In a low-acid gastric environment, oral microbiomes are more likely to colonize in the gastrointestinal tract, which may cause gastrointestinal dysfunction (10). Bile reflux, a pathologic factor driving the progression of non-CAG to CAG/GC, could promote the colonization of oral microbiota such as Veillonella, Prevotella, and Rothia in the gastric mucosa of CAG patients (18, 49). Carcinogens such as acetaldehyde and nitrosamines produced by some members of the oral microbiome may also accelerate the development of GC (21).

## CONCLUSION AND PROSPECTIVE

The review summarizes the relevant literature on the association between the oral microbiome and gastric precancerous lesions. The available literature initially concluded a potential link between the oral microbiome and gastric precancerous lesions. Furthermore, the oral microbiome may also interact with the gastric microbiomes owing to the altered gastric environment and inflammation caused by gastric precancerous lesions. Despite the results of this review showing a potential association between oral microbiome and gastric precancerous lesions, some limitations and controversies still remain.

The oral samples of existing studies are restricted, mainly containing saliva and tongue coating. However, few samples were taken from gingival crevicular fluid and subgingival plaque, which may obtain unique microorganisms associated with gastric precancerous lesions. There is also a lack of high-level evidence, such as randomized controlled trials and cohort studies. Other than the present sampling sites, potential associations between gastric precancerous lesions and microbial communities in different location of the oral cavity, such as oral mucosa and subgingival sulcus, require further investigation. In addition, large-sample observational studies or clinical trials are required to explore the role of oral microbial disorders in gastric precancerous lesions.

Based on the existing studies, the association between the oral microbiome and gastric precancerous lesions reveals significant complexities, which arises from multiple mechanisms, involving complex interactions between the altered gastric environment and microbial communities (50). When oral microbiome migrate and colonize across the stomach and intestines, its composition may vary depending on the colonization site, potentially exerting different process of carcinogenesis (50). Additionally, factors such as genetic predispositions, environmental influences, and lifestyle choices further modulate microbial distribution, contributing to the observed variability in microbial profiles associated with gastric precancerous lesions (11). These complexities lead to variations in the oral microbiome *in situ* or oral-derived microbiome found in gastric precancerous lesions, which further increases the uncertainty in the findings.
